# Development and validation of a clinical risk score for early identification of sudden acquired retinal degeneration syndrome in dogs: A retrospective case–control study

**DOI:** 10.14202/vetworld.2026.2635-2650

**Published:** 2026-08-17

**Authors:** Pasinee Sakulchatwut, Natthanet Sritrakoon, Nuanwan Rujirekasuwan, Tikamporn Teekasang, Mananya Sakulchatwut, Sirirat Niyom

**Affiliations:** 1 Ophthalmology Unit, Kasetsart University Veterinary Teaching Hospital, Bangkok 10900, Thailand.; 2 Kasetsart University Veterinary Teaching Hospital, Ratchaburi 70120, Thailand.; 3 Department of Companion Animal Clinical Sciences, Faculty of Veterinary Medicine, Kasetsart University, Bangkok 10900, Thailand.

**Keywords:** alkaline phosphatase, clinical prediction model, dogs, electroretinography, hematology, risk factors, sudden acquired retinal degeneration syndrome

## Abstract

**Background and Aim:**

Sudden acquired retinal degeneration syndrome (SARDS) is an important cause of acute, irreversible blindness in dogs and is frequently accompanied by systemic clinical and biochemical abnormalities. Despite numerous studies describing the disease, no clinically applicable prediction tool has been developed to facilitate early recognition before electroretinographic confirmation. This study aimed to identify demographic, hematological, and biochemical factors associated with SARDS and to develop and internally validate a practical clinical risk score for early identification of affected dogs.

**Materials and Methods:**

A retrospective case–control study was conducted using medical records of dogs that underwent electroretinography between January 2018 and December 2023 at the Kasetsart University Veterinary Teaching Hospital in Thailand. Fifty-three dogs with electroretinography-confirmed SARDS were compared with 219 breed-frequency-matched cataract patients exhibiting normal electroretinographic responses. Demographic characteristics, complete blood count results, and serum biochemical parameters were analyzed. Variables associated with SARDS in univariable logistic regression (p < 0.20) were entered into multivariable logistic regression using a manual purposeful selection approach. Independent predictors were incorporated into a weighted clinical risk score. Diagnostic performance was evaluated using receiver operating characteristic analysis, sensitivity, specificity, and area under the receiver operating characteristic curve (AuROC).

**Results:**

Dogs with SARDS exhibited significantly higher neutrophil counts, alkaline phosphatase (ALP) activity, plasma protein concentration, alanine aminotransferase activity, creatinine concentration, and white blood cell counts than control dogs. Multivariable logistic regression identified elevated neutrophil count (odds ratio [OR] = 8.80; p < 0.001), elevated ALP activity (OR = 6.80; p < 0.001), and increased plasma protein concentration (OR = 3.94; p = 0.004) as independent predictors of SARDS. Based on optimal cutoff values (neutrophils ≥10,525 cells/µL, ALP ≥74 U/L, and plasma protein ≥7.3 g/dL), a weighted clinical risk score ranging from 0 to 5 points was developed. A threshold score of ≥3 demonstrated good discriminative ability, with an AuROC of 0.83 (95% confidence interval: 0.76–0.90), sensitivity of 74.47%, and specificity of 79.79%, outperforming each individual laboratory parameter.

**Conclusion:**

This study identified readily available hematological and biochemical variables that are independently associated with SARDS and developed the first weighted clinical risk score for early identification of the disease in dogs. The proposed model demonstrated good diagnostic performance and may assist veterinarians in prioritizing dogs for ophthalmic evaluation and electroretinography. Although external validation in independent populations is warranted, this practical scoring system represents a valuable adjunct for improving early clinical suspicion and diagnostic decision-making in canine SARDS.

## INTRODUCTION

Sudden acquired retinal degeneration syndrome (SARDS) is widely recognized as an important cause of acute, irreversible blindness in dogs. In affected dogs, vision loss typically develops abruptly, whereas funduscopic findings are often unremarkable or only minimally abnormal at the initial presentation. Electroretinography (ERG) is therefore the cornerstone of diagnosis and typically demonstrates extinguished retinal responses consistent with severe photoreceptor dysfunction [1–3]. During ophthalmic examination, affected dogs commonly exhibit dilated pupils with minimal or absent responses to bright white light. An important diagnostic feature is the chromatic pupillary light reflex (PLR) pattern, in which the response to red light is absent, whereas the response to high-intensity blue light is preserved, reflecting dysfunction of rod- and cone-mediated pathways with retention of melanopsin-mediated retinal ganglion cell function [1, 4].

Dogs diagnosed with SARDS are predominantly middle-aged to older, with most reported cases occurring between 7 and 10 years of age. Several breeds have been consistently overrepresented, including Dachshunds, Miniature Schnauzers, Pugs, Brittany Spaniels, Bichon Frises, Maltese, American Cocker Spaniels, Beagles, Pomeranians, and Shih Tzus [1, 2, 5, 6]. Although SARDS primarily affects small-breed dogs, larger breeds may also be affected [5]. In veterinary referral ophthalmology practices where electroretinography (ERG) is readily available, SARDS represents an important cause of sudden-onset blindness [7].

Vision loss associated with SARDS progresses rapidly, and owners frequently report an abrupt onset of visual impairment. In addition to blindness, many affected dogs develop systemic clinical signs resembling hyperadrenocorticism, including polyphagia, polydipsia, polyuria, weight gain, reduced activity, and occasionally diminished olfactory function [1, 2, 8–11]. A large owner-based survey further demonstrated the rapid progression of the disease, with nearly two-thirds of affected dogs becoming blind within 2 weeks of the onset of clinical signs, while systemic abnormalities were commonly reported [3]. Although SARDS remains relatively uncommon in referral ophthalmology populations, one study identified 93 affected dogs among 5,628 dogs of the same breeds examined by a veterinary ophthalmology referral service during the study period [2]. Hematologic and biochemical abnormalities are also frequently reported, including lymphopenia, neutrophilia, proteinuria, increased liver enzyme activities, and altered plasma protein concentrations [1, 2, 8, 12].

Despite several decades of investigation, the pathogenesis of SARDS remains incompletely understood. Immune-mediated mechanisms and endocrine disturbances have both been proposed; however, neither hypothesis adequately explains the full spectrum of ocular and systemic manifestations observed in affected dogs [1, 9, 13–20]. More recently, similarities between SARDS and human ciliopathies, particularly Alström syndrome and Bardet–Biedl syndrome, have prompted consideration of SARDS as a possible acquired ciliopathy [21]. This concept has shifted research toward elucidating the underlying mechanisms of photoreceptor degeneration and associated systemic metabolic alterations. Electroretinography remains the diagnostic gold standard and typically demonstrates a flat waveform indicative of severe photoreceptor dysfunction [1–3]. Advanced retinal imaging studies have further demonstrated thinning of the outer retinal layers, along with additional structural abnormalities, supporting progressive retinal degeneration [13, 22–24]. Despite numerous therapeutic approaches, including immunosuppressive therapies, intravenous immunoglobulin administration, and other interventions, no treatment has consistently restored vision or altered disease progression [3, 15–17]. Nevertheless, long-term follow-up studies indicate that most affected dogs adapt well to permanent blindness, and euthanasia is rarely elected solely because of vision loss attributable to SARDS [25].

Although previous studies have described the demographic characteristics, clinical manifestations, and laboratory abnormalities associated with SARDS, they have primarily focused on individual risk factors rather than developing an integrated approach for early clinical recognition. Several demographic and clinicopathological variables, including breed predisposition [1, 2, 5, 6], obesity [6], neutrophilia [1, 8], and elevated liver enzyme activities [1, 2, 8, 9], have been individually associated with the disease. However, the combined predictive value of routinely available demographic, hematologic, and biochemical variables has not been systematically evaluated within a clinically applicable model readily implementable in routine veterinary practice. Consequently, veterinarians continue to rely heavily on clinical suspicion and confirmatory ERG, a specialized diagnostic procedure available primarily at referral centers. Furthermore, most published evidence has originated from North American and European canine populations, whereas comparable data from Asian populations remain scarce. This knowledge gap limits the development of evidence-based clinical decision support tools for early case identification and appropriate referral. Therefore, there is a need to develop and internally validate a practical clinical prediction model based on routinely available clinical and laboratory variables to facilitate earlier recognition of dogs at high risk of SARDS before definitive ERG confirmation.

This study aimed to identify demographic characteristics, including age, sex, neutering status, body weight, and breed, together with routinely available hematologic and biochemical variables associated with SARDS in dogs. Dogs with ERG-confirmed SARDS were compared with breed-frequency-matched dogs that demonstrated normal retinal function on ERG to identify independent predictors of the disease. Furthermore, this study sought to develop and internally validate a weighted clinical risk score based on these independent predictors to facilitate early clinical recognition, improve risk stratification, and assist veterinarians in identifying dogs that should be prioritized for comprehensive ophthalmic examination and confirmatory ERG. We hypothesized that routinely available demographic and laboratory variables would accurately discriminate dogs with SARDS from control dogs and could be integrated into a simple, robust, and clinically applicable prediction model for use in routine veterinary practice.

## MATERIALS AND METHODS

### Ethical approval

The study protocol was reviewed and approved by the Kasetsart University Institutional Animal Care and Use Committee, Kasetsart University, Bangkok, Thailand (Approval No. ACKU68-VET-039). This retrospective case–control study was conducted using archived medical records of dogs examined at the Ophthalmology Unit, Kasetsart University Veterinary Teaching Hospital, Bangkok, Thailand, between January 2018 and December 2023. All clinical examinations, laboratory investigations, and ERG procedures had been performed previously as part of routine diagnostic evaluation and clinical management. No animals were prospectively recruited, and no additional handling, restraint, sample collection, diagnostic procedures, or experimental interventions were undertaken specifically for this research. The study therefore involved no additional risk, discomfort, or alteration of treatment for the enrolled dogs.

Owner consent for the original clinical examinations, diagnostic testing, ERG, and use of clinical information for institutional research purposes was obtained in accordance with the hospital’s standard clinical and ethical procedures. The investigators accessed only information necessary to address the study objectives, and all records were reviewed retrospectively under institutional authorization. Identifying information about dogs and their owners was removed or coded prior to analysis to protect confidentiality and privacy. Data were handled securely and reported only in aggregate form, ensuring that individual animals, owners, and clinical records could not be identified. The study was conducted in accordance with the institutional requirements for animal welfare, research integrity, confidentiality, and responsible use of veterinary medical records.

### Study period and location

This retrospective study was conducted using medical records of dogs presented to the Ophthalmology Unit at the Kasetsart University Veterinary Teaching Hospital in Bangkok, Thailand. Medical records of dogs examined between January 2018 and December 2023 were reviewed.

### Study design

A retrospective case–control study was performed using a 6-year diagnostic registry of dogs that underwent ERG. Cases consisted of dogs diagnosed with SARDS based on a history of acute blindness and bilaterally extinguished ERG responses. The control group comprised breed-frequency-matched cataract patients with normal ERG responses who were examined during the same period, serving as a reference population with objectively confirmed preserved retinal function.

To minimize selection bias and analytical variability, all cases originated from a single veterinary referral ophthalmology service, and all hematologic and biochemical analyses were performed using standardized diagnostic protocols in the Central Laboratory of Kasetsart University Veterinary Teaching Hospital. This centralized laboratory approach minimized interlaboratory analytical variation. In addition, because of the hospital population's regional demographics, the study included a substantial representation of the native Thai Bangkaew breed, enabling characterization of this population within the SARDS cohort. Because performing ERG in clinically healthy dogs is neither ethically nor practically justified, cataract patients with normal ERG responses were considered an appropriate surrogate control population.

### Case and control selection

Dogs were included in the SARDS group if they had a clinical history of acute blindness, absent menace responses and dazzle reflexes despite intact PLR, unremarkable or only minimally abnormal funduscopic findings at presentation, and bilaterally extinguished ERG responses. All eligible cases were examined during the study period.

The control group consisted of cataract patients with normal ERG responses examined during the same period. A breed-frequency-matched control population was generated by selecting dogs from the same breeds represented among SARDS cases, thereby ensuring comparable breed distributions between groups without individual matching.

Dogs were excluded from the control group if they had concurrent systemic diseases despite normal ERG waveforms before cataract surgery. Dogs with abnormal ERG waveforms before cataract surgery, including reduced-amplitude or extinguished responses, were also excluded, as were dogs with vision loss attributable to other ocular or neurologic disorders, including cortical blindness, optic neuritis, progressive retinal atrophy, glaucoma, retinal detachment, or ocular trauma.

**Figure 1:**
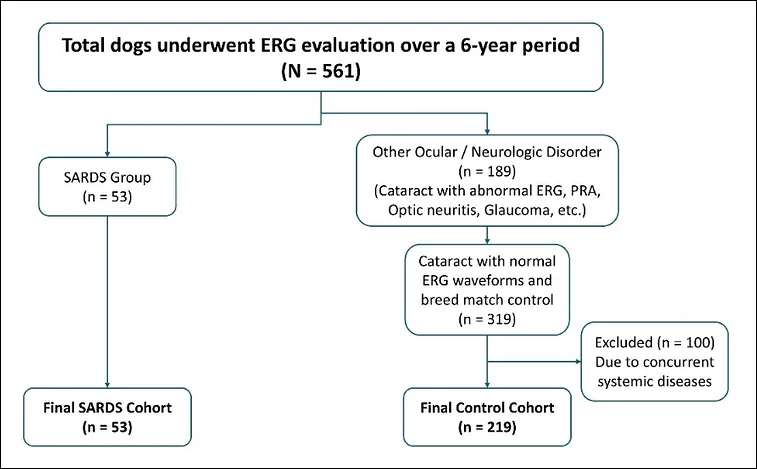
Flow diagram illustrating the retrospective screening process, exclusion criteria, and final enrollment of dogs in the SARDS and control groups over the 6-year study period.

Among the 319 cataract patients with normal ERG responses identified during the study period, dogs with concurrent systemic diseases were excluded. These conditions included metabolic and endocrine disorders (diabetes mellitus; n = 70), hepatobiliary diseases (n = 25), including gallbladder sludge, cholestasis, hepatitis, and hepatic lipidosis, blood parasitic infections (n = 3), systemic hypertension (n = 1), and protein-losing nephropathy (n = 1). Following application of these exclusion criteria, 219 clinically stable dogs remained eligible for statistical analysis (Figure 1).

### Data collection

Medical records were reviewed to obtain signalment (age, sex, neutering status, body weight, and breed) and complete blood count (CBC) and serum biochemical data. Hematologic variables included hemoglobin (HGB), hematocrit (HCT), red blood cell count (RBC), mean corpuscular volume (MCV), mean corpuscular hemoglobin (MCH), mean corpuscular hemoglobin concentration (MCHC), white blood cell count (WBC), neutrophil count, lymphocyte count, monocyte count, eosinophil count, basophil count, and platelet count. Serum biochemical variables included plasma protein, blood urea nitrogen (BUN), creatinine, alanine aminotransferase (ALT), alkaline phosphatase (ALP), total protein, and albumin.

Body weight was categorized according to breed-specific reference weight ranges established by the American Kennel Club breed standards [26]. Because body condition score (BCS) was not consistently available in the medical records, breed-specific reference body weights were used as a surrogate indicator of body weight status. Dogs weighing above the upper reference limit for their breed were classified as overweight, whereas those weighing below the lower reference limit were classified as underweight. Dogs were also categorized into two age groups (<6 years and ≥6 years) based on previous SARDS studies, which consistently reported that most affected dogs were older than 6 years and used this threshold for clinical stratification [7].

The CBC was performed using an automated hematology analyzer (Sysmex XN-1000™, Sysmex, Mundelein, IL, USA). Serum biochemical analyses were performed using an IL Lab 650 Chemistry System (Diamond Diagnostics, Holliston, MA, USA). All reference intervals used in this study were those established by the Central Laboratory of Kasetsart University Veterinary Teaching Hospital for the corresponding diagnostic instruments.

### Statistical analysis

Descriptive statistics were used to summarize demographic characteristics and laboratory variables for both study groups. Continuous variables were assessed for normality using the Shapiro–Wilk test and are presented as mean ± standard deviation (SD) for normally distributed data or median (interquartile range [IQR]) for non-normally distributed data. Categorical variables are presented as frequencies and percentages.

Missing predictor data were managed using a complete-case analysis approach, whereby dogs with missing values for variables included in a given regression model were excluded from that analysis.

Univariable logistic regression analysis was performed to evaluate the association between each explanatory variable and SARDS status. Variables with p < 0.20 were considered eligible for inclusion in the multivariable logistic regression model to avoid excluding potentially important predictors. To minimize model overfitting, the number of candidate variables was restricted according to an events-per-variable ratio of at least 10. A manual purposeful selection strategy was subsequently used to derive the final multivariable model.

Multicollinearity among predictor variables was assessed using chi-square tests for categorical variables, pairwise correlation coefficients for continuous variables, variance inflation factors (VIFs), and tolerance values. When substantial multicollinearity was identified, only one of the correlated variables was retained in the final model.

The diagnostic performance of significant predictors was evaluated using the area under the receiver operating characteristic curve (AuROC), sensitivity, specificity, and corresponding 95% confidence intervals (CI). Optimal cutoff values were determined using Youden's index to maximize the combined sensitivity and specificity. Positive predictive value (PPV) and negative predictive value (NPV) were also calculated.

A weighted clinical risk score was subsequently developed using the variables retained in the final multivariable logistic regression model. Clinically relevant predictors with statistically significant associations were selected for inclusion. Continuous variables were dichotomized using both established clinical reference intervals and optimal cutoff values obtained from receiver operating characteristic (ROC) curve analysis to improve clinical applicability. Regression coefficients from the final multivariable model were divided by the smallest coefficient and rounded to the nearest integer to generate weighted scores for each predictor. Individual scores were summed to obtain the total clinical risk score, with higher scores indicating a greater probability of SARDS.

The predictive performance of the derived risk score was evaluated in the development cohort using the AuROC, sensitivity, specificity, PPV, NPV, and positive likelihood ratio. The optimal total score cutoff was determined using Youden's index. All statistical analyses were performed using jamovi statistical software (Version 2.3; The jamovi Project, Sydney, Australia).

To our knowledge, this study represents the first application of a weighted point-based clinical risk score for SARDS derived using regression coefficient weighting. This transparent and clinically interpretable approach provides a practical tool for estimating the probability of SARDS using routinely available clinical and laboratory variables before confirmatory ERG testing.

## RESULTS

### Diagnostic distribution and clinical characteristics of dogs with SARDS

During the 6-year study period, ERG findings from 561 dogs were reviewed. The diagnoses included cataract (70.23%; 394/561), SARDS (9.45%; 53/561), progressive retinal atrophy (7.31%; 41/561), cortical blindness (6.42%; 36/561), optic neuritis (5.70%; 32/561), glaucoma (0.53%; 3/561), day blindness (0.18%; 1/561), and congenital retinopathy (0.18%; 1/561).

Among the 53 dogs diagnosed with SARDS, the age at onset of blindness ranged from 3 to 13 years, and the mean age at diagnosis was 9.07 ± 2.30 years. The SARDS group comprised 24 females and 29 males. Crossbreeds were the most frequently represented dogs (30.2%; 16/53), followed by Thai Bangkaew dogs (18.9%; 10/53), Poodles (9.4%; 5/53), Miniature Schnauzers (9.4%; 5/53), Pomeranians (9.4%; 5/53), Pugs (7.5%; 4/53), Shih Tzus (5.7%; 3/53), Chihuahuas (3.8%; 2/53), French Bulldogs (3.8%; 2/53), and Yorkshire Terriers (1.9%; 1/53). The principal systemic clinical signs were polyphagia (47.2%; 25/53), polyuria (45.3%; 24/53), and polydipsia (45.3%; 24/53).

Complete vision loss developed within <2 weeks in 36 dogs and within ≥2 weeks in 17 dogs. Menace responses and dazzle reflexes were absent in all 53 dogs. PLRs were present in 7 dogs, incomplete in 23 dogs, and absent in 23 dogs. Funduscopic examination revealed normal fundi in 34 dogs and abnormalities in 19 dogs, including decreased retinal vessel caliber, retinal vascular attenuation, and tapetal hyperreflectivity. The interval between the onset of blindness and laboratory and ERG evaluation was <2 weeks in 12 dogs, approximately 1 month in 24 dogs, 2 months in 11 dogs, and ≥3 months in 6 dogs.

### Demographic and laboratory characteristics

Several breed-related differences were identified between the SARDS and control groups (Table 1). Thai Bangkaew dogs were more frequently represented in the SARDS group than in the control group (18.9% vs. 3.7%; p < 0.001). Binomial logistic regression indicated that Thai Bangkaew dogs had 6.13-fold greater odds of SARDS than all other breeds combined (95% CI = 2.29–16.44; p < 0.001), suggesting a potential breed predisposition within the study population.

Miniature Schnauzers (9.4% vs. 1.4%; p = 0.007), Pomeranians (9.4% vs. 2.3%; p = 0.022), and Pugs (7.5% vs. 1.4%; p = 0.023) were also more frequently represented in the SARDS group. Conversely, Poodles were less frequently represented in the SARDS group than in the control group (9.4% vs. 26.0%; p = 0.014). No significant differences were detected for crossbreeds, Shih Tzus, Chihuahuas, French Bulldogs, or Yorkshire Terriers.

Neutering status differed significantly between the groups (p = 0.005), with neutered dogs accounting for 75.5% of the SARDS group and 54.8% of the control group. Body weight also differed significantly between the groups (p = 0.001). However, neither age category (p = 0.075) nor sex (p = 0.640) differed significantly.

Dogs with SARDS had significantly higher WBC and neutrophil counts and significantly lower lymphocyte counts than control dogs (all p < 0.001). Plasma protein concentration, creatinine concentration, ALT activity, ALP activity, and albumin concentration were also significantly higher in the SARDS group. In particular, median ALP activity was 463.9 U/L in dogs with SARDS compared with 107.1 U/L in control dogs (p < 0.001). No significant between-group differences were observed in the remaining erythrocyte indices, platelet count, total protein concentration, or BUN concentration (Table 1).

**Table 1:** Demographic characteristics and laboratory variables of dogs with SARDS and control dogs.

**Patient characteristic**	**Control group (n = 219)**	**SARDS group (n = 53)**	**p-value**
Breedᵃ			
Crossbreed	70 (32.0%)	16 (30.2%)	0.803
Thai Bangkaew	8 (3.7%)	10 (18.9%)	<0.001
Poodle	57 (26.0%)	5 (9.4%)	0.014
Miniature Schnauzer	3 (1.4%)	5 (9.4%)	0.007
Pomeranian	5 (2.3%)	5 (9.4%)	0.022
Pug	3 (1.4%)	4 (7.5%)	0.023
Shih Tzu	28 (12.8%)	3 (5.7%)	0.155
Chihuahua	24 (11.0%)	2 (3.8%)	0.129
French Bulldog	15 (6.8%)	2 (3.8%)	0.414
Yorkshire Terrier	6 (2.7%)	1 (1.9%)	0.726
Sex			0.640
Female	107 (48.9%)	24 (45.3%)	
Male	112 (51.1%)	29 (54.7%)	
Neutering status			0.005
Neutered	120 (54.8%)	40 (75.5%)	
Intact	98 (44.7%)	12 (22.6%)	
Unknown	1 (0.5%)	1 (1.9%)	
Age			0.075
<6 years	39 (17.8%)	4 (7.5%)	
≥6 years	180 (82.2%)	49 (92.5%)	
Body weight (kg)			0.001
Crossbreed	12.80 (4.00–35.00)	20.47 (5.60–40.00)	
Thai Bangkaew	24.77 (19.80–39.40)	26.16 (19.84–32.90)	
Poodle	6.35 (3.10–14.00)	10.80 (6.20–17.90)	
Miniature Schnauzer	11.13 (7.50–17.50)	11.13 (7.50–17.50)	
Pomeranian	3.80 (2.90–4.80)	6.25 (1.60–9.30)	
Pug	12.33 (12.20–12.50)	11.11 (7.30–12.75)	
Shih Tzu	6.30 (3.40–10.00)	7.30 (5.60–10.10)	
Chihuahua	3.88 (1.70–7.60)	3.40 (2.40–4.40)	
French Bulldog	13.76 (9.90–18.60)	14.20 (14.20–14.20)	
Yorkshire Terrier	3.00 (2.30–3.80)	2.40 (2.40–2.40)	
Laboratory variables			
HCT (35–57%)	45.47 ± 4.89	45.28 ± 5.56	0.803
HGB (11.9–18.9 g/dL)	16.03 ± 1.84	15.86 ± 2.02	0.553
RBC (4.95–7.87 × 10⁶ cells/µL)	6.88 ± 0.83	6.90 ± 0.86	0.879
MCV (66–77 fL)	66.25 ± 3.58	65.94 ± 3.42	0.570
MCHC (32.0–36.3 g/dL)	35.26 ± 1.25	34.90 ± 1.62	0.086
MCH (21.0–26.2 pg)	23.35 ± 1.38	23.00 ± 1.38	0.106
WBC (5,000–14,100 cells/µL)	9,949 (7,815–11,785)	12,300 (8,640–13,900)	<0.001
Neutrophil count (2,900–12,000 cells/µL)	7,043 (5,298–8,583)	9,995 (6,728–11,970)	<0.001
Lymphocyte count (400–2,900 cells/µL)	1,910 (1,323–2,313)	1,248 (855–1,677)	<0.001
Monocyte count (100–1,400 cells/µL)	443 (230–600)	546 (288–680)	0.058
Eosinophil count (0–1,300 cells/µL)	573 (237–770)	471 (200–660)	0.149
Basophil count (0–140 cells/µL)	10 (0–10)	15 (0–10)	0.434
Platelet count (211–621 × 10³ cells/µL)	315 (227–381)	294 (214–360)	0.230
Plasma protein (6.0–8.0 g/dL)	7.4 (7.0–8.0)	7.9 (7.4–8.4)	<0.001
Total protein (5.4–7.5 g/dL)	7.0 (6.4–7.3)	7.1 (6.7–7.6)	0.432
BUN (8–28 mg/dL)	21.6 (15.0–26.0)	21.1 (15.0–23.3)	0.766
Creatinine (0.5–1.7 mg/dL)	1.0 (0.8–1.1)	1.1 (0.9–1.3)	0.001
ALT (10–109 U/L)	52.3 (30.0–59.0)	97.8 (43.0–132.0)	<0.001
ALP (1–114 U/L)	107.1 (30.3–98.5)	463.9 (93.8–462.0)	<0.001
Albumin (2.3–3.1 g/dL)	3.2 (3.0–3.4)	3.3 (3.2–3.6)	0.044

ᵃEach breed was compared with all other breeds combined. Values are presented as n (%), mean ± SD, or median (IQR), as appropriate. HCT = hematocrit; HGB = hemoglobin; RBC = red blood cell count; MCV = mean corpuscular volume; MCHC = mean corpuscular hemoglobin concentration; MCH = mean corpuscular hemoglobin; WBC = white blood cell count; BUN = blood urea nitrogen; ALT = alanine aminotransferase; ALP = alkaline phosphatase; SD = standard deviation; IQR = interquartile range; SARDS = sudden acquired retinal degeneration syndrome.

### Univariable analysis of factors associated with SARDS

Univariable logistic regression was performed to identify factors potentially associated with SARDS (Table 2). Variables with p < 0.20 were considered for multivariable analysis, subject to the requirement of at least 10 outcome events per predictor. Based on statistical significance, clinical relevance, data completeness, collinearity assessment, and the events-per-variable criterion, neutering status, high body weight, high neutrophil count, high plasma protein concentration, and high ALP activity were selected for the initial multivariable model.

**Table 2:** Univariable logistic regression analysis of factors associated with SARDS in dogs.

**Variable**	**Estimate (β)**	**SE**	**OR (95% CI)**	**Missing values**	**p-value**
Age ≥6 years versus <6 years	−0.976	0.549	0.377 (0.128–1.105)	0 (0%)	0.075
Female versus male	0.144	0.307	1.154 (0.632–2.108)	0 (0%)	0.640
Neutered versus intact	−1.001	0.356	0.367 (0.183–0.738)	2 (0.74%)	0.005
Body weight				0 (0%)	
High versus normal	1.196	0.363	3.305 (1.623–6.733)		<0.001
Low versus normal	0.162	0.428	1.176 (0.508–2.721)		0.705
HGB				2 (0.74%)	
High versus normal	−0.061	0.657	0.941 (0.260–3.410)		0.926
Low versus normal	1.074	0.927	2.928 (0.475–18.025)		0.247
HCT, low versus normal	1.058	0.926	2.880 (0.469–17.694)	0 (0%)	0.253
RBC				2 (0.74%)	
High versus normal	−0.026	0.482	0.974 (0.379–2.508)		0.957
Low versus normal	NE	NE	NE		NE
MCV, low versus normal	−0.203	0.314	0.816 (0.442–1.509)	2 (0.74%)	0.518
MCHC				2 (0.74%)	
High versus normal	−1.289	0.620	0.276 (0.082–0.929)		0.038
Low versus normal	1.326	1.424	3.766 (0.231–61.338)		0.352
MCH				2 (0.74%)	
High versus normal	0.843	1.236	2.322 (0.206–26.167)		0.495
Low versus normal	1.066	0.593	2.903 (0.907–9.286)		0.072
WBC				0 (0%)	
High versus normal	1.000	0.401	2.718 (1.239–5.959)		0.013
Low versus normal	NE	NE	NE		NE
Neutrophil count				6 (2.21%)	
High versus normal	2.398	0.530	11.000 (3.891–31.095)		<0.001
Low versus normal	NE	NE	NE		NE
Lymphocyte count				6 (2.21%)	
High versus normal	NE	NE	NE		NE
Low versus normal	0.625	1.235	1.867 (0.166–21.022)		0.613
Monocyte count				6 (2.21%)	
High versus normal	1.430	1.424	4.178 (0.256–68.075)		0.315
Low versus normal	−0.480	0.561	0.619 (0.206–1.858)		0.392
Eosinophil count, high versus normal	−1.297	1.045	0.273 (0.035–2.120)	6 (2.21%)	0.215
Basophil count, high versus normal	1.479	1.423	4.388 (0.270–71.374)	6 (2.21%)	0.299
Platelet count				0 (0%)	
High versus normal	NE	NE	NE		NE
Low versus normal	0.589	0.371	1.802 (0.871–3.730)		0.112
Plasma protein, high versus normal	1.758	0.380	5.803 (2.754–12.225)	7 (2.57%)	<0.001
BUN				3 (1.10%)	
High versus normal	−0.553	0.469	0.575 (0.230–1.441)		0.238
Low versus normal	0.964	0.928	2.651 (0.425–16.170)		0.299
Creatinine				0 (0%)	
High versus normal	2.166	1.235	8.720 (0.775–98.069)		0.079
Low versus normal	NE	NE	NE		NE
ALT				1 (0.37%)	
High versus normal	2.348	0.422	10.465 (4.580–23.915)		<0.001
Low versus normal	NE	NE	NE		NE
ALP, high versus normal	2.181	0.359	8.857 (4.384–17.896)	34 (12.50%)	<0.001
Total protein				112 (41.18%)	
High versus normal	0.796	0.442	2.218 (0.932–5.278)		0.072
Low versus normal	NE	NE	NE		NE
Albumin				104 (38.34%)	
High versus normal	0.808	0.394	2.244 (1.037–4.854)		0.040
Low versus normal	NE	NE	NE		NE

SE = standard error; OR = odds ratio; CI = confidence interval; NE = not estimable because of a zero cell count; HGB = hemoglobin; HCT = hematocrit; RBC = red blood cell count; MCV = mean corpuscular volume; MCHC = mean corpuscular hemoglobin concentration; MCH = mean corpuscular hemoglobin; WBC = white blood cell count; BUN = blood urea nitrogen; ALT = alanine aminotransferase; ALP = alkaline phosphatase; SARDS = sudden acquired retinal degeneration syndrome.

### Multivariable analysis of independent factors associated with SARDS

The initial multivariable logistic regression model included neutering status, high body weight, high neutrophil count, high plasma protein concentration, and high ALP activity (Table 3). High neutrophil count, high plasma protein concentration, and high ALP activity remained significantly associated with SARDS. Neutering status (p = 0.074) and high body weight (p = 0.128) did not remain statistically significant and were excluded during manual purposeful selection.

**Table 3:** Initial multivariable logistic regression model of factors associated with SARDS in dogs.

**Variable**	**Estimate (β)**	**SE**	**OR (95% CI)**	**p-value**	**VIF**
Neutering status	−0.816	0.457	0.442 (0.181–1.083)	0.074	1.030
High versus normal body weight	0.720	0.474	2.054 (0.812–5.197)	0.128	1.021
High versus normal neutrophil count	2.025	0.639	7.579 (2.165–26.530)	0.002	1.011
High versus normal plasma protein	1.261	0.502	3.530 (1.321–9.435)	0.012	1.021
High versus normal ALP	1.691	0.405	5.424 (2.451–12.005)	<0.001	1.026

SE = standard error; OR = odds ratio; CI = confidence interval; VIF = variance inflation factor; ALP = alkaline phosphatase; SARDS = sudden acquired retinal degeneration syndrome.

The final multivariable model identified high neutrophil count, high ALP activity, and high plasma protein concentration as independent factors associated with SARDS (Table 4). High neutrophil count was associated with 8.80-fold greater odds of SARDS (OR = 8.795; 95% CI = 2.630–29.406; p < 0.001). High ALP activity was associated with 6.80-fold greater odds (OR = 6.796; 95% CI = 3.170–14.567; p < 0.001), whereas high plasma protein concentration was associated with 3.94-fold greater odds (OR = 3.937; 95% CI = 1.560–9.937; p = 0.004). VIF values were close to 1.0, indicating no meaningful multicollinearity among the retained predictors.

**Table 4:** Final multivariable logistic regression model of factors associated with SARDS in dogs.

**Variable**	**Estimate (β)**	**SE**	**OR (95% CI)**	**p-value**	**VIF**
High versus normal neutrophil count	2.174	0.616	8.795 (2.630–29.406)	<0.001	1.003
High versus normal plasma protein	1.370	0.472	3.937 (1.560–9.937)	0.004	1.003
High versus normal ALP	1.916	0.389	6.796 (3.170–14.567)	<0.001	1.005

SE = standard error; OR = odds ratio; CI = confidence interval; VIF = variance inflation factor; ALP = alkaline phosphatase; SARDS = sudden acquired retinal degeneration syndrome.

### Diagnostic performance of individual predictors

ROC curve analysis was used to evaluate the diagnostic performance of the three independent predictors (Table 5). ALP activity demonstrated the greatest individual discriminative ability, with an AuROC of 0.82 (95% CI = 0.75–0.89). At the optimal cutoff of ≥74 U/L, ALP activity had a sensitivity of 83.33% and a specificity of 66.84%.

Neutrophil count and plasma protein concentration each had an AuROC of 0.70. A neutrophil cutoff of ≥10,525 cells/µL provided high specificity (91.67%) but relatively low sensitivity (42.00%). In contrast, a plasma protein cutoff of ≥7.3 g/dL provided higher sensitivity (78.00%) but lower specificity (54.88%).

**Table 5:** Diagnostic performance of individual predictors of SARDS based on ROC curve analysis.

**Optimal cutoff determined using Youden’s index**	**AuROC** ** (95% CI)**	**Sensitivity (95% CI)**	**Specificity (95% CI)**
Neutrophil count ≥10,525 cells/µL	0.70 (0.62–0.79)	42.00% (28.19%–56.79%)	91.67% (87.15%–94.99%)
Plasma protein ≥7.3 g/dL	0.70 (0.62–0.79)	78.00% (64.04%–88.47%)	54.88% (47.97%–61.66%)
ALP ≥74 U/L	0.82 (0.75–0.89)	83.33% (69.78%–92.52%)	66.84% (59.66%–73.49%)

ROC = receiver operating characteristic; AuROC = area under the receiver operating characteristic curve; CI = confidence interval; ALP = alkaline phosphatase; SARDS = sudden acquired retinal degeneration syndrome.

### Development of the clinical risk score

A weighted clinical risk score was developed from the final multivariable model using the optimal cutoff values for neutrophil count, plasma protein concentration, and ALP activity (Table 6). A neutrophil count ≥10,525 cells/µL was assigned 2 points, plasma protein concentration ≥7.3 g/dL was assigned 1 point, and ALP activity ≥74 U/L was assigned 2 points. Values below these thresholds were assigned 0 points. The resulting total score ranged from 0 to 5 points.

**Table 6:** Components of the clinical risk score derived from the final multivariable logistic regression model.

**Variable**	**Regression coefficient (β)**	**OR (95% CI)**	**p-value**	**VIF**	**Score**
Neutrophil count (cells/µL)					
<10,525	Reference	Reference		1.003	0
≥10,525	2.17	8.80 (2.63–29.41)	<0.001		2
Plasma protein (g/dL)					
<7.3	Reference	Reference		1.003	0
≥7.3	1.37	3.94 (1.56–9.94)	0.004		1
ALP (U/L)					
<74	Reference	Reference		1.005	0
≥74	1.92	6.80 (3.17–14.57)	<0.001		2

OR = odds ratio; CI = confidence interval; VIF = variance inflation factor; ALP = alkaline phosphatase.

### Diagnostic performance of the clinical risk score

The overall diagnostic performance of the three-factor clinical risk score was evaluated and visualized using an ROC curve (Table 7 and Figure 2). The combined score achieved an AuROC of 0.83 (95% CI = 0.76–0.90), exceeding the discriminative performance of each individual predictor.

The optimal cutoff for identifying dogs at increased risk of SARDS was ≥3 points. At this threshold, the score had a sensitivity of 74.47%, specificity of 79.79%, positive predictive value of 47.95%, NPV of 92.59%, and positive likelihood ratio of 3.68.

**Table 7:** Diagnostic performance of the three-factor clinical risk score for SARDS.

**Score range**	**Optimal cutoff**	**AuROC** **(95% CI)**	**Sensitivity ** **(95% CI)**	**Specificity ** **(95% CI)**	**PPV (95% CI)**	**NPV (95% CI)**	**LR+ (95% CI)**
0–5 points	≥3 points	0.83 (0.76–0.90)	74.47% (59.65%–86.06%)	79.79% (73.33%–85.28%)	47.95% (39.85%–56.15%)	92.59% (88.41%–95.34%)	3.68 (2.65–5.12)

SARDS = sudden acquired retinal degeneration syndrome; AuROC = area under the receiver operating characteristic curve; CI = confidence interval; PPV = positive predictive value; NPV = negative predictive value; LR+ = positive likelihood ratio. The three predictors included in the score were neutrophil count, plasma protein concentration, and ALP activity.

### Distribution of clinical risk scores

The distribution of total clinical risk scores was compared between dogs with SARDS and control dogs using complete-case data (Table 8). The analysis included 47 dogs with SARDS and 188 control dogs. Scores were concentrated at the lower end of the scale among control dogs, with 33.0% scoring 0 points and 26.1% scoring 1 point. Only 2.1% of control dogs had scores of 4 or 5 points.

In contrast, dogs with SARDS were concentrated at the higher end of the score distribution. Overall, 74.5% of dogs with SARDS had scores ≥3 points, including 36.2% with 3 points and 29.8% with 5 points. This separation in score distributions further supported the discriminative ability of the three-factor scoring system.

**Table 8:** Distribution of the three-factor clinical risk score among dogs with sudden acquired retinal degeneration syndrome (SARDS) and control dogs.

**Total risk score (points)**	**Control group (n = 188)**	**SARDS group (n = 47)**
0	62 (33.0%)	2 (4.3%)
1	49 (26.1%)	5 (10.6%)
2	39 (20.7%)	5 (10.6%)
3	34 (18.1%)	17 (36.2%)
4	1 (0.5%)	4 (8.5%)
5	3 (1.6%)	14 (29.8%)

Values are presented as n (%). The total risk score ranged from 0 to 5 points and was calculated using complete-case data for neutrophil count, plasma protein concentration, and ALP activity. Dogs with missing data for any of these variables were excluded, comprising 31 control dogs and 6 dogs with SARDS.

## DISCUSSION

### Principal findings

The present study identified several routinely available clinical and laboratory variables that were significantly associated with SARDS in dogs. Increased neutrophil count, plasma protein concentration, and ALP activity remained independently associated with SARDS in the final multivariable model. These findings support the concept that SARDS is accompanied by systemic inflammatory, metabolic, and hepatobiliary alterations rather than being restricted to the retina. Importantly, this study developed and internally evaluated a practical clinical risk score based entirely on routine laboratory variables that are widely accessible in veterinary practice. The study also provides geographic novelty as one of the largest SARDS cohorts reported from Asia and the first from Thailand, with a distinctive breed distribution that included substantial representation of the native Thai Bangkaew dog.

### Hepatic enzyme alterations

Consistent with previous reports, dogs with SARDS had significantly increased liver enzyme activities, particularly ALP. Although ALT activity was also significantly higher in dogs with SARDS during univariable analysis, median ALT values in both groups remained within the institutional reference interval, suggesting that the difference may have limited clinical relevance. In contrast, ALP activity in dogs with SARDS markedly exceeded the upper reference limit, indicating a more pronounced biochemical abnormality.

Because ALT and ALP were collinear, only one hepatic enzyme was retained in the multivariable model to improve model stability. ALP was selected because of its stronger association with SARDS, greater clinical interpretability, and previously established relevance to the disease, particularly in relation to increased corticosteroid-induced ALP isoenzyme activity and concurrent vacuolar hepatopathy [1, 2, 8, 9, 27–30]. These hepatic alterations may reflect chronic endogenous corticosteroid exposure, exogenous corticosteroid administration, or dysregulation of the hypothalamic–pituitary–adrenal axis, all of which have been described in dogs with SARDS. Although increased ALP activity is nonspecific, its frequent occurrence in dogs with acute blindness may serve as a clinical warning sign and support the need for further ophthalmic investigation.

### Plasma protein concentration

Increased plasma protein concentration was independently associated with SARDS. This finding is consistent with previous evidence indicating persistent hyperproteinemia after SARDS diagnosis. One review reported that plasma protein concentrations may remain elevated for up to 1 year after disease onset [1]. In addition, a comparative study found significantly higher plasma protein concentrations in dogs with SARDS than in healthy controls, with mean values of 7.9 and 6.7 g/dL, respectively (p < 0.001) [12].

In contrast, another study evaluating hemostatic parameters found no statistically significant difference in plasma protein concentrations between dogs with SARDS and controls, although 4 of 5 affected dogs had increased concentrations [31]. The mechanism underlying hyperproteinemia in SARDS remains unclear. Possible explanations include chronic inflammation, immune activation, hemoconcentration, altered hepatic protein synthesis, or changes in protein metabolism associated with endocrine dysfunction. The present findings further support the concept that SARDS involves systemic processes beyond retinal degeneration; however, the retrospective design precludes conclusions regarding causality.

### Hematologic alterations and systemic stress

Dogs with SARDS had significantly higher neutrophil counts and lower lymphocyte counts than control dogs. This pattern is compatible with a stress leukogram and may be mediated by increased circulating glucocorticoid concentrations. Cortisol promotes neutrophil demargination, prolongs neutrophil survival, inhibits neutrophil apoptosis, and contributes to lymphocyte redistribution and apoptosis. Chronic physiological stress may also alter canine neutrophil phenotype, surface-receptor expression, and leukocyte survival dynamics [32–35]. The observation of lymphopenia is consistent with the effects of increased circulating glucocorticoids during systemic stress [36].

Previous studies have also reported neutrophilia in dogs with SARDS, with prevalences of 42.86% in one small cohort and 18% in another [8, 31]. The recurrence of this finding across studies suggests that neutrophilia may be a reproducible hematologic feature of the disease, although its magnitude may vary according to disease stage, concurrent illness, treatment history, and the laboratory reference intervals used.

The combination of increased neutrophil and decreased lymphocyte counts would also be expected to increase the neutrophil-to-lymphocyte ratio. In veterinary medicine, an elevated neutrophil-to-lymphocyte ratio has been investigated as a readily accessible biomarker of systemic inflammation, physiological stress, and prognosis in several canine diseases, including mammary tumors and chronic inflammatory enteropathies [37, 38]. Although the neutrophil-to-lymphocyte ratio was not directly evaluated in the present study, the observed leukocyte pattern provides additional evidence that dogs with SARDS may experience marked systemic stress and immune dysregulation.

### Breed distribution and potential predisposition

The breed distribution in the present cohort demonstrated both similarities to international reports and features specific to the regional population. Miniature Schnauzers and Pugs were significantly more frequent among dogs with SARDS than among controls, consistent with previous reports identifying these small breeds as potentially predisposed populations. Their predisposition may be associated with genetic, metabolic, or endocrine characteristics that overlap with proposed mechanisms of SARDS.

A particularly important epidemiological observation was the high representation of Thai Bangkaew dogs, which accounted for 18.9% of the SARDS group compared with 3.7% of the control group. Thai Bangkaew dogs had approximately sixfold higher odds of SARDS than all other breeds combined. Because this native breed is concentrated primarily in Thailand, its potential association with SARDS has not been described in international studies. This finding emphasizes the importance of considering regional breed demographics when investigating disease predisposition. Nevertheless, referral patterns, breed popularity, and the relatively small number of Thai Bangkaew dogs should be considered before concluding that a true genetic predisposition exists. Multicenter studies and genomic investigations are required to confirm this association.

Pomeranians were also more frequently represented in the SARDS group, whereas Poodles were less frequently represented. However, breed-specific estimates based on small sample sizes may be unstable and should be interpreted with caution. The observed differences may reflect true predisposition, regional breed prevalence, cataract-related referral patterns within the control group, or a combination of these factors.

### Clinical risk score and diagnostic utility

To our knowledge, no previously published clinical prediction model or point-based risk score for SARDS is available. Earlier studies have largely described clinical manifestations and individual associations, including obesity, metabolic abnormalities, hyperadrenocorticism-like signs, toxicological exposures, and altered hemostatic profiles [2, 5–7, 31]. Although specialized assessments such as thromboelastography and anti-retinal antibody testing may provide pathophysiological information [20, 31], they are not routinely available in most primary veterinary practices and were not designed as practical screening models.

The three-factor score developed in this study incorporated neutrophil count, plasma protein concentration, and ALP activity. These variables are routinely measured during the initial diagnostic evaluation of dogs with acute blindness. The combined score achieved an AuROC of 0.83, indicating good discrimination between dogs with SARDS and controls. At the optimal cutoff of ≥3 points, the score had a sensitivity of 74.47% and a specificity of 79.79%.

The score also had a high NPV of 92.59% in the study population. This finding indicates that dogs scoring below the cutoff were unlikely to have SARDS within this referral cohort. However, predictive values depend strongly on disease prevalence and may differ substantially across populations with higher or lower SARDS prevalence. Therefore, the NPV should not be interpreted as a fixed probability applicable to all clinical settings. External validation is required before the score can be recommended as a definitive rule-out tool.

The PPV was 47.95%, indicating that fewer than half of the dogs scoring ≥3 points had SARDS in this dataset. Consequently, a high score should increase clinical suspicion and support referral for specialist ophthalmic assessment but should not be considered diagnostic. Similarly, the positive likelihood ratio of 3.68 represents a moderate increase in the probability of disease rather than conclusive confirmation.

The score is not intended to replace ERG, which remains the definitive diagnostic method for demonstrating severe generalized retinal dysfunction. Instead, it may assist clinicians in prioritizing referral, counseling owners, and distinguishing dogs that warrant urgent ophthalmic evaluation. Because no treatment has been consistently demonstrated to restore vision or arrest retinal degeneration in SARDS, the score should not be used as the sole basis for initiating unproven immunomodulatory or neuroprotective treatment before diagnostic confirmation. Its most appropriate role is as a clinical decision support and triage tool.

### Control group considerations

Selecting an appropriate control group for SARDS studies is methodologically challenging. Ideally, controls would comprise dogs at risk of developing SARDS but without the disease and with objectively confirmed normal retinal function. However, ERG is rarely performed in clinically normal dogs without ocular disease, making such recruitment ethically and practically difficult.

The present study therefore used cataract patients with normal ERG responses as controls. This approach provided objective confirmation of preserved retinal function and ensured that controls underwent the same diagnostic procedure as SARDS cases. Nevertheless, cataract patients may differ from the general canine population in age, breed distribution, metabolic status, and referral patterns. These differences could introduce selection bias and may partly explain the lower frequency of certain breeds in the SARDS group, particularly breeds commonly predisposed to cataract.

The exclusion of cataract patients with concurrent systemic diseases reduced confounding from conditions that could independently alter hematologic and biochemical variables. However, excluding 100 of 319 dogs with normal ERG responses may also have created a comparatively healthy control group and increased the apparent strength of associations between systemic abnormalities and SARDS. Therefore, the reported ORs should be interpreted as associations relative to this selected cataract-control population rather than to the general dog population.

### Geographic and referral-related limitations

The study population was derived from a single university referral hospital in Thailand. The distinctive regional breed distribution, particularly the representation of Thai Bangkaew dogs, may limit generalizability to populations in other countries. The diagnostic performance of the risk score may differ in Western cohorts or regions with substantially different breed compositions, disease prevalences, and clinical referral practices.

Referral bias is also likely because dogs evaluated at a university hospital may have more acute, severe, or diagnostically complex disease than dogs managed in primary care. Owners attending a referral center may also differ in their ability or willingness to pursue specialized diagnostic testing. Consequently, the associations and predictive performance observed in this cohort require validation in primary care and multicenter populations.

### Additional limitations

The retrospective design resulted in incomplete documentation and missing data for several variables. BCS was not consistently available, preventing direct assessment of adiposity. Breed-specific reference weights based on American Kennel Club standards were therefore used as a surrogate measure. However, breed standard weights do not directly measure fat mass, may not be appropriate for crossbreeds, and may incorrectly classify heavily built or unusually small dogs. Accordingly, the observed association between high body weight and SARDS should be interpreted cautiously.

Endocrine testing was available for only a subset of dogs, which prevented a comprehensive evaluation of hyperadrenocorticism, thyroid dysfunction, diabetes mellitus, and other hormonal abnormalities. Clinical signs such as polyphagia, polyuria, and polydipsia were also incompletely documented in control dogs, limiting direct comparison of systemic manifestations between groups.

Complete-case analysis reduced the sample size used to develop and evaluate the final score. The risk score analysis included 47 of 53 dogs with SARDS and 188 of 219 control dogs. If missingness was associated with disease severity or clinical decision-making, complete-case analysis may have introduced additional bias. Furthermore, the score was developed and evaluated in the same cohort. The reported AuROC therefore reflects apparent or internally evaluated performance and may be optimistic when applied to new populations.

Although the final model demonstrated low VIF values and a McFadden R² of 0.258, these measures do not rule out residual confounding, selection bias, or overfitting. The number of SARDS cases was modest, and some breed-specific analyses involved very small cell counts. In addition, dichotomization of continuous variables improved clinical simplicity but reduced information and may have decreased predictive efficiency.

### Clinical implications and future research

The three-factor risk score provides a potentially cost-effective method to support the clinical evaluation of dogs with acute blindness. Unlike ERG, the required variables are derived from routine hematologic and biochemical testing available in most veterinary clinics. A score ≥3 points may justify prioritizing ophthalmic referral and ERG, whereas a lower score may reduce suspicion within populations similar to the present cohort. Nevertheless, the score must be interpreted together with the history, ophthalmic examination, PLRs, funduscopic findings, and differential diagnoses.

Prospective external validation is the most important next step. Future studies should evaluate the score in geographically diverse populations, primary care settings, and independent referral cohorts. Validation should include calibration, discrimination, decision-curve analysis, and assessment of predictive values across different disease prevalences. Bootstrap validation or cross-validation may also provide more reliable estimates of optimism during model refinement.

Future models should incorporate standardized BCS, detailed endocrine profiles, duration of blindness, systemic clinical signs, medication history, and ophthalmic variables. Evaluation of the neutrophil-to-lymphocyte ratio may also clarify whether it provides additional predictive value beyond absolute neutrophil count. Larger datasets could support investigation of non-linear relationships, interaction terms, and machine-learning methods. However, any more complex model should be compared with the current three-variable score to determine whether improvements in predictive performance justify reduced interpretability and increased implementation complexity.

Overall, this study provides preliminary evidence that a simple combination of neutrophil count, plasma protein concentration, and ALP activity can support risk stratification in dogs suspected of having SARDS. Although retrospective, single-center, and internally evaluated, the model represents a foundational step toward evidence-based clinical decision support for SARDS and warrants prospective multicenter validation.

## CONCLUSION

This study identified key demographic and clinicopathological factors associated with SARDS in dogs and developed the first clinically applicable, weighted risk score for early identification of affected patients using routinely available clinical data. Dogs with SARDS were significantly more likely to exhibit increased neutrophil counts, elevated ALP activity, and higher plasma protein concentrations than control dogs. Multivariable logistic regression confirmed these three variables as independent predictors of SARDS, and integrating them into a simple point-based scoring system yielded good discriminative performance, with an AuROC of 0.83, sensitivity of 74.47%, and specificity of 79.79%. Importantly, the combined risk score outperformed each individual laboratory parameter, supporting the value of a multivariable approach for clinical decision-making.

A major strength of this study is the use of ERG-confirmed SARDS cases and ERG-confirmed controls with preserved retinal function, minimizing disease misclassification. The study also evaluated a comprehensive panel of routinely available demographic, hematological, and biochemical variables using a robust multivariable modeling strategy. Furthermore, this is the first study to derive a weighted clinical risk score for SARDS and to demonstrate its diagnostic performance in an Asian canine population.

The retrospective case–control design limits causal inference and is subject to inherent selection bias. The use of cataract patients with normal ERG, rather than healthy dogs, as the control population may also affect the generalizability of the findings. In addition, the prediction model underwent only internal validation within a single referral institution, and external validation in independent populations was not performed.

The present study demonstrates that elevated neutrophil count, ALP activity, and plasma protein concentration are significant independent predictors of SARDS in dogs and can be combined into a practical clinical risk score with good diagnostic performance. While ERG remains the gold standard for definitive diagnosis, the proposed scoring system is a valuable adjunctive tool to support early clinical suspicion and referral. These findings provide a foundation for future validation studies and help improve the early recognition and clinical management of canine SARDS.

## DATA AVAILABILITY

The supplementary data can be made available from the corresponding author upon request.

## GENERATIVE AI DECLARATION

The authors declare that generative artificial intelligence (AI) tools were used solely to improve language, grammar, and readability during manuscript preparation. All scientific content, data analysis, interpretation of results, and conclusions were developed and verified by the authors. The authors take full responsibility for the accuracy, integrity, and originality of the work presented, and no AI tool was listed as an author.

## AUTHORS’ CONTRIBUTIONS

PS: Conceived and designed the study, coordinated the project, performed the statistical analysis, and drafted the manuscript. MS: Assisted with data collection and database management. NS, NR, and TT: Performed the electroretinography examinations and contributed clinical data. SN: Supervised the project, critically reviewed the study design and statistical analyses, and revised the manuscript for important intellectual content. All authors have read and approved the final version of the manuscript.
